# Gold and Carbon-Based Nano-theranostics: An Overview on the Developments and Applications for Cancer Phototherapy

**DOI:** 10.34172/apb.2022.071

**Published:** 2021-10-02

**Authors:** Ashay Manisha Shailendrakumar, Vamshi Krishna Tippavajhala

**Affiliations:** Department of Pharmaceutics, Manipal College of Pharmaceutical Sciences, Manipal Academy of Higher Education, Manipal, Karnataka, India.

**Keywords:** Nano-theranostics, Gold nanoparticles, Carbon nanotubes, Photothermal therapy, Photodynamic therapy

## Abstract

Nano-theranostics (NTs) are versatile nanomaterials, explored in the current scenario of cancer therapy. A nano-theranostic material alone can diagnose and generate a therapeutic effect. Various materials have been explored for their NT action like gold and carbon-based material. The photon-based cancer theranostics has grabbed the attention of researchers due to their localized and trigger activated effect. NTs have shown a promising result in pre-clinical and clinical studies. The current review illustrates the meticulous efforts conducted by researchers across the globe to innovate and explore the photon-based cancer NT platforms of gold and carbon with their application in cancer therapy.

## Introduction

The cancer is known for its aggressive nature, complexity and heterogeneity. It is the second leading cause of death globally. The World Health Organization›s (WHO’s) “Cancer profile 2020” illustrated that among 18 million globally reported cancer cases, around 9.6 million have died in 2018.^1^ The number of cancer cases are expected to rise (double) by 2040.^2^

Conventional therapy shows low solubility, low selectivity and less specificity. Due to low selectivity to cancer cells, conventional therapy may cause toxicity to normal cells. Henceforth these cannot act effectively on the cancer cells. It may result in low therapeutic outcomes, resulting in an in-effective treatment.^3^ These limitations of conventional therapy have given a positive push to researchers, for developing new treatment approaches for cancer for e.g., photon, immune, gene-based, target deliveries and nanomedicine etc.^3,4^

Plethora of literature on research carried in the nanomedicine field has shown an effective treatment of cancer with targeted deliveries.^4^ Nano-theranostics (NTs) are one among them possessing a dual property to act as a therapeutic agent and a diagnostic probe.^5^ They showed effective mitigation and improved the clinical outcome of the cancer treatment.^5^

The focused research and development of NTs have shown an excellent therapeutic outcome in *ex vivo* and *in vivo* studies. The versatile nature of NTs has drawn attention due to their enormous diagnostic and therapeutic capacity. Moreover, the multimodality, effective transport capacity, ability to combat resistance, and compatibility with a wide range of materials (actives e.g., siRNA, biological drugs, proteins, etc) has made NTs a remarkable strategy in cancer therapy.^4,5^

Typically, a nano-theranostic consists of a photosensitizer with or without an active material. Upon injection of the NTs to the subject, they enter at the target through passive or active targeting as illustrated in Table 1.

**Table 1:** Types of targeting with nano-theranostics

**Type of targeting**	**Mechanisms/impact on cancer cells**
Passive targeting	High proliferation rate of tumour cell creates deformed vasculature and capillary fenestration. It extravasates and retains the nanomaterial in tumour cells.
Active targeting	Nanomaterial accumulates and favours interaction with the cancer cell recognizing specifically over expressing molecules.

**Figure 1:**
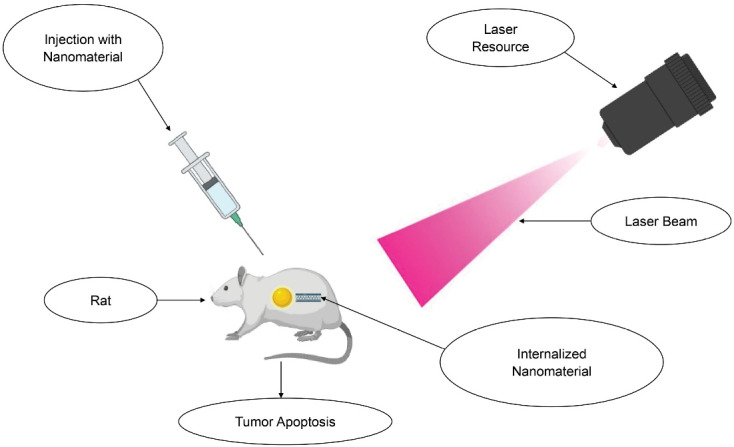
Targeted delivery of nano-theranostics

At the target, an externally illuminated laser activates the administered material. It is collectively represented in Figure 1.

**Figure 2:**
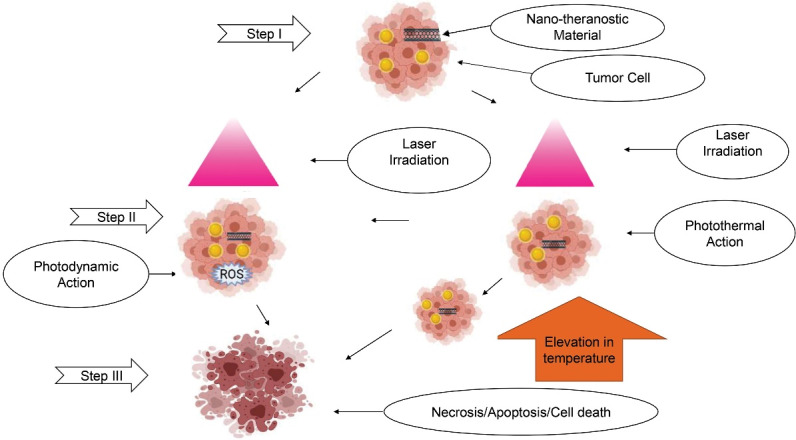
Mechanism of action of photon based Nano-Theranostics

Upon activation, NT acts either by photothermal therapy (PTT) or photodynamic therapy (PDT). The NTs for PDT generate a reactive oxygen species (ROS). It causes death of cancer cells (demonstrated in Figure 2) by the mechanisms as illustrated in Table 2.

**Table 2:** Mechanism of Nano-theranostics therapies exerted on cells

	**Action**	**Temperature Elevation**	**Mechanisms**	**Ref**
Photo thermal therapy	NA		L-SPR: It is a collective oscillation. It is mediated with light scattered absorption. The absorbed light (as an energy) is dissipated as local heat. The light excites the free electrons (present in the internalized NTs) converts light into heat.	^6,7^
Photo dynamic therapy	NA		The photosensitizer in the material generates the ROS, like singlet oxygen (1O2), free radicals and peroxides on excitation of NT material with NIR light. The ROS is cytotoxic in nature. It causes irreversible damage to tumour tissues, called as photodynamic effect.	^8,9^
Hyperthermia	Lysosomal damage	41°C to 46°C	Increases sensitivity of tumour without affecting the protein synthesis. It activates the apoptosis by expressing or inducing proteins	^7^
Thermal ablation	Cell necrosis	46°C to 70°C	The elevated temperature modifies biomolecules; denatures proteins, to lose their activity; and melts the lipid	^7^
Nano photo thermolysis	Dual action necrosis with oncosis	NA	Temperature elevation results in micro explosion at the surface of NTs with no heat transfer to the surrounding tissue. It causes an irreversible cell damage by expulsion from the nanoparticle surface or generated ultrasonic shock	^7^
LANTCET [Laser-activated nano-thermolysis as cell elimination technology]	Activation by reaction of vapor and plasmon resonance	NA	The vapor bubbles generated due to plasmon resonance effect of NTs, disrupts the cancer cell membrane and cytoskeleton through mechanical forces	^7^

Abbreviations: L-SPR, localized surface plasmon resonance; ROS, reactive oxygen species; NA, not applicable; NIR, near infra-red.

 Such excellent and promising mechanisms by NTs made scientists curious to explore them for effectiveness in the cancer treatment for the therapeutic, diagnostic, and neoplastic ability. Of the various NTs, metal-based NTs have received more attention, due to their notable effectiveness in pre-clinical and clinical stages of drug design with a feasible candidature. The metal-based NTs are easily functionalized and are inert. The metal-based NTs contain a core (gold, silver, iron, copper, platinum, gallium, and cobalt) and shell (containing polymeric or any other material) supporting the core section. Gold, iron, and copper-based NTs are at the forefront for research. The carbon-NTs (quantum dots, fullerenes, carbon nanotubes, and graphene) have also received major attention recently. They show good electronic, mechanical, optical, and thermal properties with versatile functionalization.

The NTs are known to be synthesized using various methods. It includes chemical methods like the Turkevich method, pyrolysis, spray methods and reduction etc. They are also reported to be synthesized by electrochemical method and latest method like pulverization method as enlisted in Table 3.

**Table 3:** Synthesis methods of nano-theranostics

**Material**	**Methods**	**Ref**
Gold	Turkevich method	^10,11^
	Brust-Schiffrin method	^12^
	Galvanic replacement	^13,14^
	Cobalt sacrifice	^15^
	Seeding	^12^
	Desalting method	^16^
Carbon based material	Electric arc discharge	^17^
	Laser ablation	^18^
	Chemical vapor deposition	^18^
	Flame synthesis	^19^
	Spray pyrolysis	^18^
	Pulverization	^20^
GO	Exfoliation	^21^
R-GO	Reduction	^21^

Abbreviations: GO, Graphene oxide; R-GO, Reduced graphene oxide

 The current review summarizes the vastly explored NTs like gold, carbon nanotubes (CNT) and graphene with morphologies and case studies in photothermal and photodynamic therapies.

## Gold based nano-theranostics

**Figure 3:**
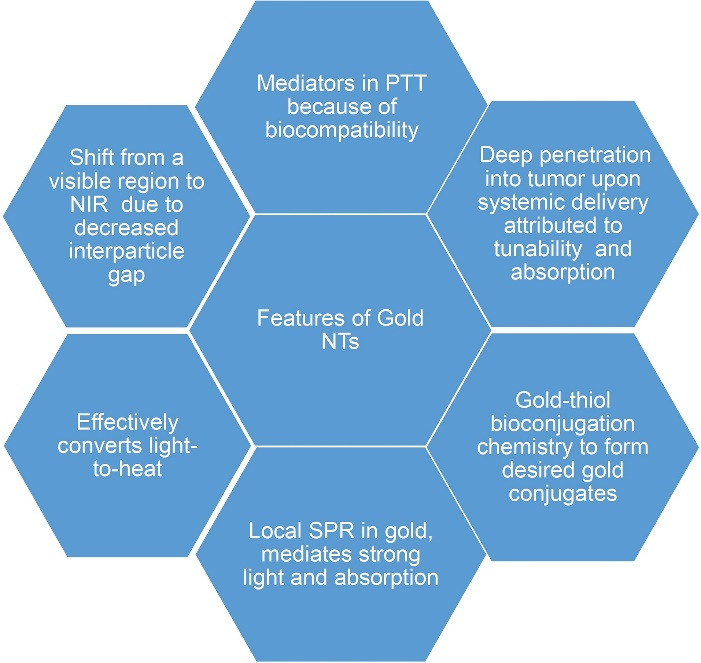
Features of gold nano-theranostics

Gold nanoparticles (GNPs) are extensively explored for cancer treatment. Gold (Au) is a choice for NTs, because of its appreciable chemo-physical stability, easy functionalization, synthesis, optical tunability and biocompatibility.^3^ The various properties and excellent features of gold have made gold-based NT a versatile tool (Figure 3).

**Figure 4:**
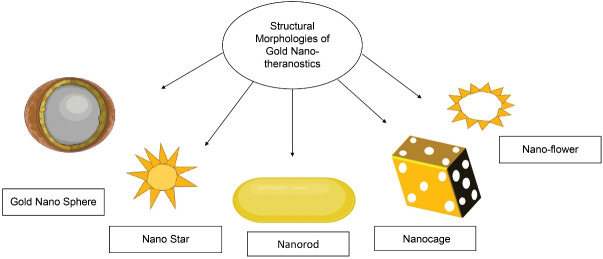
Structural morphologies of the gold nano-theranostics

The low toxicity and excellent bio-compliance of GNPs, have made them ideal for the theranostic application.^9^ They are synthesized in various shapes and sizes ranging from 2nm to 100 nm with different shapes and morphologies (Figure 4). Their properties vary, according to size and shape.^22^

## Gold-based nano-theranostics for cancer photo thermal therapy

GNPs are non-invasive multifunctional tools with unique and excellent features.^23^ They load a wide range of active materials owing to their high surface-to-volume ratio.^23^ The integration of the GNPs, for targeted delivery and diagnostic tools, has overcome the constraints related to conventional cancer treatment.^23^ GNPs have ensured the real-time monitoring of co-delivered diagnostic and therapeutic molecules at the target.^23^ Targeted imaging with combined delivery of anticancer agent and gold has enhanced the retention of GNP at the site.^23^ They are validated agents with synergistic effects for the different treatments (viz. efficient drug loading, PTT, antisense DNA), by single conjugation.^23^

## Gold nano-stars (GNS)

It is a star-shaped GNP. For a GNS, its absorption band depends on core diameter, spike number, spike length and sharpness. The sharpness of spike tips promotes the shift in the peak absorption (NIR region). The core of a GNS exhibits a short wavelength band in the mid-visible region.^12^ The plasmon peaks of GNS are attributed to the modes associated with the inner core and branch tips.^12^ Surface plasmon resonance (red-shift) of GNS changes with its increasing size.^12^ The simultaneous red-shift by a long plasmon band increases the spike length of GNS.^12^ The electronic (E)-field of GNS is enhanced by peak sharpness.^12^ The local surface plasmon resonance (L-SPR) in the branches, confines at the tip edge and excited by incident Electric (E)-field components.^12^

###  Case studies

Chen and colleagues have explored the application of GNS for the treatment of breast and lung cancer. It was featuring a triple purpose of imaging with combined PTT and chemo-therapy mode. The functionalization of GNS with peptide c-RGD (complex peptide of arginine-glycine-aspartic acid) enhanced its absorption capacity. For the tumour imaging, the authors conjugated GNS-RGD with MPA (an indocyanine green derivative). For the purpose of tumour ablation, they conjugated doxorubicin (DOX) separately.^11^ The authors additionally evaluated GNS conjugates. The *in vivo* targeting capacity of MPA conjugates (2 mL with 10 mg/kg) was evaluated in mice models induced with MCF-7 and MDA-MB-231 (integrin αvβ3-overexpressing) cells. The MPA conjugates showed a good binding and distribution implicating its good diagnostic capacity. The In-vivo PTT evaluation of GNS conjugates was carried on S180 tumor-bearing mice. Upon irradiation, GNS conjugates (Au-MPA, Au-cRGD-MPA, Au-DOX and Au-cRGD-DOX at concentration 50 µg/mL) achieved a temperature up to 43ºC. Upon irradiation, GNS targeted subcellular integrin αvβ3-overexpressing tumors and showed their destructive effects with PTT characterization in different cell cultures (MCF-7, MDA-MB-231 and Bel-7402).^11^ After the therapy, microscopic observation of a lesion showed a significant morphological change with tumour apoptosis. Upon post-treatment evaluation of GNS in the cancer cells showed maximum apoptosis in MDA-MB-231 and Bel-7402 cells with aid of IR. The Au-cRGD-DOX showed 1.3 times higher inhibition compared to plain GNS and drug. Overall, the GNS conjugates demonstrated an enhanced anti-tumour effect with triplet combination compared to a single material.^11^

In another study, plasmonic (pl) GNS, were found applicable and useful for cancer phototherapy. The application was specific to light-mediated and cell-targeted surface-enhanced scattering. To investigate the effectiveness of plasmonic GNS, Song et al developed a multifunctional plasmonic GNS-RGD protein conjugate. The authors applied them for the diagnosis and detection of lung cancer. The conjugates were intended to act in the two different ranges of NIR I and NIR II, for SERS imaging. The authors carried in-vitro studies in the A549 human cells. The plasmonic-PTT at two NIR conditions showed an effective ablation.^24^ The conjugates exerted a PTT effect at 785 nm (Condition1/NIR I) upon NIR irradiation, with 95.7% and 80.6% absorption. Irradiation elevated the temperature of the surroundings by 7.1ºC at 1 minute and 16˚C after 10 minutes.^24^ The irradiation at 1064 nm (Condition 2/NIR II) showed an elevation up to 14.7ºC with a 13% heat conversion capacity.^24^ *In vivo* evaluation of GNS on A549 cells showed good specificity and low cell viability.^24^ Condition 2 showed that GNS required a low concentration and low irradiation power with enhanced cytotoxicity and 88%-97% viability to A549 cells at a decreased concentration (0.1 mg/mL) with good internalization.^24^ The *in vitro* photothermal action of plasmonic GNS on A549 culture at 785 nm, 390 mW/cm^2^, for 6 minutes showed a good anti-cancer activity compared to untreated ones with a lower activity.^24^

## Gold nano-cages (GNCs)

GNC is a hollow NT approach that shows a photothermal transducer capacity. It is well known for its excellent therapeutic applications. GNCs are proven well suitable for PTT with encapsulation and controlled release of an active agent (with the aid of NIR).^25^ The specialty of the product includes an in-process control of L-SPR positioning, with monitoring of chloroauric acid addition or titration in the process. GNCs can be precisely tuned in any wavelength from 600–1200 nm with L-SPR at 800nm. GNCs absorb NIR radiation, five times more compared to conventional photo-sensitizers.^25^

###  Case study

Chan et al evaluated the effectiveness of the GNC for cancer.^25^ They functionalized thiol conjugated-GNC with methoxy-terminated poly ethylene glycol (m-PEG), which aided the accumulation of GNC in tumour with passive targeting. The authors investigated GNC for the in-vivo PTT effect on bilateral-tumours induced with glioblastoma (U87wtEGFR) cells. GNCs maintained a good concentration in blood circulation. GNCs accumulated in the tumour and showed enhanced penetration and retention effect.^25^ GNC›s at a concentration of 1011 particles/mL, effectively enhanced the temperature. Additionally, the GNCs showed reduced tumour activity (detected with leaky vasculature and the cancer cell damage and pyknosis and karyolysis) on glioblastoma cells induced mouse model with positron emission tomography for tumour metabolism (Compared with the positive control group administered with saline). The biodistribution study of GNC indicated an effective uptake of the nanocages, with accumulation at the periphery of tumours causing irreversible tumour damage.^25^

## Gold nano-flowers (GNFs)

The nanoflowers are among the core structures, which show an obvious advantage over the conventional morphologies related to NIR absorption.^26^ The larger size of nanoflower shows low toxicity due to its low uptake. Nanoflowers have exhibited a good photothermal effect even at lower doses.^26^

###  Case study

Novel conjugates of GNF were constructed by Han et al using a tree-type surfactant (C18N3) by surface crystal growth.^26^ They formulated Nanoflowers in four different sizes i.e., 115 nm, 126 nm, 146 nm, 153 nm. They were later evaluated *in vitro* and in-vivo on HeLa cells and female Balb/c murine model (induced with HeLa cells) respectively.^26^ Upon induction of laser, GNFs effectively controlled tumour and ablated HeLa cells at 70°C.^26^ The best *in vitro* activity at elevated temperature was showed by GNF of 153 nm. The 20 days *in vivo* treatment, abolished the tumour with no obvious presence of side effects and low toxicity compared to the plain group showing no presence of tumour lesion.^26^ Overall, the study showed that control on the size of GNF can easily tune the absorption peak from visible to the NIR region.^26^

## Gold nano-spheres (GNSPs)

GNSPs are the most preferred for the studies among the GNPs, because of the simplified quick synthesis and easy conjugation.^27^ Their SPR depends on the thickness, hybrid plasmon, presence of free electrons on the surface (number), and phase delay.^27^ The tuning of GNSP enhances resonance, scattering, and absorption. GNSP shows maximum tuning in 800 nm-1200 nm. They easily absorb light from 500 to 600 nm.^27^ Both hollow and solid GNSP have applications in PTT.^28^ GNSPs can be easily conjugated with a wide range of therapeutic agents e.g. DOX, proteins, and peptide. The hollow GNSP offers an internal space for drugs and enzymes loading, which acts as a delivery vehicle.^27^ They are formulated by galvanic replacement, desalting and chemical synthesis.^28^

###  Case studies

An investigation was carried by You et al who designed a hollow gold nanosphere (HGN) for breast cancer, with a feature of dual function as a drug carrier with cancer destruction.^28^ The modified HGN showed 63% drug loading (1.7 g DOX).^28^ Upon laser induction, the DOX -HGN showed a PTT conversion with a triggered fast release (pH + NIR combination) out of HGN compared to the PEG conjugate of DOX-HGN and the groups without treatment.^28^ The presence of an acidic environment enhanced the DOX release from HGN, compared to alkaline pH. The *in vitro* evaluation on MDA-MB-231 cells showed a significant cancer ablation 1.2(HGN-DOX) and 1.24(PEG-HGN-DOX) times higher compared to the plain HGN who showed up to 70% ( > 69%) ablation of the cells (5.9 µg/mL) and HGN+NIR showing 40% cell killing.^28^

GNSPs have also shown their effectivity with human cervix cancer wherein, Mendoza-Nava et al developed GNSP for cervix cancer. Authors modified GNSP by peptide conjugation.^29^ The *in vitro* evaluation of GNSP on HeLa cells (octreotide v/s citrate gold complex) revealed a signiﬁcant temperature elevation up to 50ºC by both conjugates.^29^ The study also revealed that the presence of octreotide (peptide) conjugates signiﬁcantly decreased cell viability compared to the gold-citrate complex.^29^ The study showed the suitability of a GNSP-octreotide complex for treating cervical cancer.^29^

Combination with tumour necrosis factor (TNF): Similar to the previous study, Shao et al developed a TNF alpha containing PEG conjugates of GNSPs.^30^ An *in vivo* photothermal tumour ablation capacity (on SCK cells on A/J mice) of GNSP was comparatively tested with standalone lasers of 532 nm and 690 nm individually on test groups (test groups: the plain laser, combined GNSP-TNF, and GNSP-PEG conjugate). Post-treatment the animals in the 6th group-containing GNSP with TNF irradiated at 690 nm survived and showed reduced tumour weight.^30^ The irradiation of GNSP with a laser increased temperature, which induced thermal expansion, explosion and fragmentation. This mechanism also formed acoustic waves, which generated nanobubbles and microbubbles which ablated the cancer cells.^30^ Post-treatment evaluation of GNSP conjugates showed a deep penetration of drug in the tissue making GNSP an effective delivery vehicle. The formed nanobubbles, physically damaged tumour cells by an explosion. Irradiation also generated small size and drug fragments.^30^

## Gold nano-rods (GNRs)

Like GNSPs, GNRs have also been deeply studied for melanoma, fast PTT and many more applications. They are reported to be synthesized by electrochemical, nucleation and photochemical methods. The key features of GNR like prolong stability, capacity to act as a contrast agent and effective delivery of therapeutic molecules makes GNR the best among the GNPs.^31,32^ The length to width ratio of GNR is responsible for its absorption capacity (range 3-6.6). The cancer cells effectively take up GNRs ranging in the size 10-40 nm.^27^ They offer a dual SPR due to its short and long axis, i.e. transversally at 500–550 nm and longitudinally at 650–850 nm. GNR shows a maximum absorption peak at 800 nm with effective transduction.^9^ Scientists have proved that GNRs are efficient in targeted PTT for Melanoma and fast therapies etc.^31^

###  Case studies

Targeting on overexpressing gene: Peng et al formulated GNR-chitosan conjugates of monoclonal antibody for targeted therapy of neuroblastoma.^33^ The presence of monoclonal antibody in the conjugates simplified its transport into the cells by antibody-endocytosis. The *in vitro* evaluation of GNR conjugates on neuroblastoma cells (SH-SY5Y-GD2- cell and stNB-V1-GD2+cell) was carried out using MTT assay. It showed selective destruction of the GD2+ overexpressing cells by GNR-probes.^33^

Melanoma cancer: The treatment of melanoma with GNR containing PEI against transfected HSP70 (Heat Shock Protein) was reported by Andersson et al.^34^ The efficiency of the GNRs was evaluated on the HeLa cells by the *in vitro* testing and *in vivo* studies in mice injected with B16 melanoma cells respectively.^34^ An important finding in the *in vitro* study was the highest level of EGFP (HSP expressed green fluorescence) expression showed the lowest toxicity. It was achieved by using approximately 1012 GNR and 10 pulses at 50 J/cm^2^, with a 30-millisecond pulse length. In general, the cells are driven to increase the production of EGFP with increased heating up to a particular threshold. At the threshold point, cells damaged rapidly and reduced the expression and eventually viability. The studies showed that GNR with PEI demonstrated a site-directed, heat-inducible gene reporter expression (GFP) with low cell viability.^34^

Another study on GNR-PEG conjugates was carried by Yao et al. It showed specificity and high accumulation with controlled cell (A549) death (20 seconds) with low laser throw-put (9.3 mW).^35^ Irradiation of GNR caused field enhancement with SPR coupling.^35^ It aided the temperature elevation and induced the death of a single cell in a short time (GNR concentration 26pM) with 98% viability.^35^ GNRs also showed synergistic ablation of BART cells, without affecting healthy cells.^36^ For breast cancer, GNRs showed effective temperature elevation. The 20-day treatment ablated the cancer cells and stopped the regrowth of cancer.^37^ Similar to the previous study, Siloxane based GNRs were also found effective in lung cancer.^38^

## Photodynamic therapy with GNPs

GNP intended for PDT contains a photosensitizer, tuneable in the 700-1100 nm range, for targeting a deeply located tumour.^39^ Upon incidence of laser on the NTs, the light gets converted to heat. It aids the electron in the gold to reach in an excited state which in turn activates the photosensitizer and generates the ROS.

###  Case study

A study was reported by Lkhagvadulam et al on GNPs for purpose of PDT. Authors developed GNP conjugates of purpurin-18-N-Methyl-D Glucamine, in the size range 5-11 nm, 27-44 nm and 50-70 nm.^40^ A549 cell line was used as a cell culture for evaluation of the *in vitro* activity of GNP.^40^ The GNP-conjugates showed better activity compared to the plain molecule. The nano-clusters with 60nm size showed the highest *in vitro* PDT (compared to 11 nm, 27 nm and70 nm) action (amount of ROS generated) due to the good transport compared to other conjugates and plain photosensitizer.^40^

## Combined therapies with GNPs

 Both PTT and PDT have been explored together as a combination therapy. Most of the gold NTs intended for this purpose contains multiple agents or material in a single conjugate. These have been proven effective for controlling cancer with the aid of chemical or immune agents, biomolecules etc. either as a single or two in a one GNP probe.

###  Case studies

An investigation was carried out by Wang and colleagues on a multipurpose GNS for treating breast cancer using both PTT and PDT. The PDT evaluation on the MCF-7 breast cancer cells showed an effective ROS generation resulting in DNA damage. The *in vivo* investigation of GNS on Balb/C mice cancer model (S-180 cells) showed effective energy to heat conversion, with a temperature increase of 11ºC.^41^ Compared to the blank group (Injected with normal saline) GNS showed effective cell apoptosis. After the *in vivo* assessment, it was concluded that cell death was observed up to 57%. Among the observed 57% cell apoptosis, 27% of the cell death was found with PDT and the remaining 30% due to PTT.^41^ Additionally, the X-ray imaging with GNS confirmed the cell death, proving the capability of the conjugates to be developed as a diagnostic agent.^41^

Protein-based conjugates for PDT: Though the PDT is advanced therapy, but it turns ineffective when the availability of oxygen is low due to poorly developed blood vessels. It is responsible for higher peroxide generation. It results in a hypoxic condition, which is detrimental to the PDT treatment. Considering these issues, Lin Zhang and colleagues developed an intelligent GNS for synergistic action of PTT & PDT. The probes showed various good features such as high attenuation and good penetrable depth. Loading of Ce6 (chlorine e6) coated with the mesoporous silica, alleviated the solubility problem of Ce6, improved its stability, and prevented toxicity caused by Ce6. Probes also prevented the premature release of the Ce-6 by the photothermal mechanism i.e., silicon served as a switch off and switch on button for release.^42^ The *in vitro* combined PDT/PTT evaluation of conjugates on HeLa, and MCF 7 cells showed cell death upon increased temperature (60ºC).^42^ The comparative evaluation of the probes (GNS-CE-silicon conjugated with PEG, DSPE and RGD separately) showed the highest activity with DSPE conjugates. A CT scan imaging of the lesion showed an accurate targeting of conjugates with a highly expressive gene at the site.^42^ Similar results were observed with the *in vivo* evaluation of the probes on Balb/C mice induced with cancer cells.

Treatment of multi-resistant (MDR) colorectal cancer: Recently resistance of cancer to the drug is becoming a cause of concern in the treatment. Scientists across the globe are working rigorously on combating drug resistance. In the context of the same, a nano-theranostic probe was developed by Jiang et al. They formulated GNS conjugates of mesoporous silica with a pH-responsive poly-histidine (PHIS) polymer for combined therapy. The authors formulated two conjugates of GNS containing PHIS with tocopheryl polyethylene glycol succinate (TPGS) and PHIS+TPGS+DOX. The polymer aided the intracellular accumulation of GNR with endo/lysosome escape transport. The presence of PEG and tocopherol on the surface realized intracellular drug retention by inhibiting P-glycoprotein. Upon irradiation, GNRs showed a faster PTT conversion with drug release. Overall the *in vitro* (SW620/Ad300 plain v/s formulation) and *in vivo* (SW620/Ad300 induced mice) evaluation showed that GNRs containing DOX+TPGS were effective with PTT and pH-responsive combination and overcame the MDR cancer.^43^ The post-therapy evaluation of the tumours showed that GNS containing DOX was 10 times more active compared to plain drug and the test group without laser (comparison over tumour volume).

Another study in the context of biomolecule-aided GNS was conducted by Choi and colleagues. They carried a comparative investigation of the GNR conjugated biomolecule for PDT and PTT. The authors evaluated the comparative performance of the GNR-conjugated antibody and aptamer separately on A431and MCF7 induced mice model (groups antibody-GNR: A431 and MCF7, aptamer-GNR: A431 and MCF7). In both *in vitro* and *in vivo* evaluation, aptamer-based conjugates showed an effective uptake by cells compared to antibody-GNR. Additionally, an aptamer-GNR ablated of high amount cell compared to antibody GNR.^44^ In A431 cells the inhibition zone was observed wider compared to MCF7 cells.^44^

Symphony-based PDT: Treatment of brain cancer is relatively challenging compared to the rest of the cancers. Henceforth, a symphony approach for the treatment of brain cancer was explored by Liu and colleagues using plasmonic GNS. The study was evaluated in C57BL/6 mice induced with CT-2A glioma cells. Primarily, GNS coated material was accumulated at the target. Irradiation of accumulated plasmonic GNS at the site converted light to heat with a cell ablation. Subsequently, the second treatment of induced hyperthermia was carried separately with an antibody (PD-L1). This showed benefit to an antibody with improved access to T cell enabling. T cell enabling, elicited a synergistic and effective anti-tumour action. The combination of the material reduced tumour volume 20 times than probes containing GNS irradiated with INR. The treatment also rejected a rechallenge with memorized anticancer response, showing a safe treatment for aggressive Glioblastoma.^45^

## Carbon-based nano-theranostics

**Figure 5:**
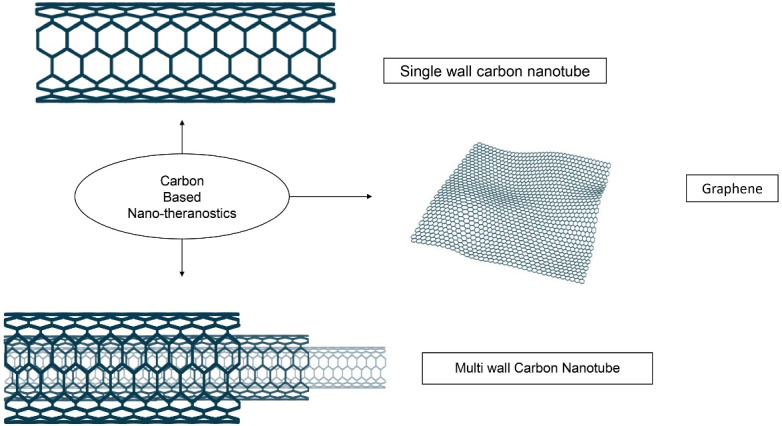
Structural morphologies of the carbon based nano-theranostics

Carbon-based NTs also showed an effective cancer treatment. The carbon-based materials are composed of carbon bonds joined together in a thick form. The basic structure contains a honeycomb lattice of carbon atoms. It can be modified and converted to diversified geometries. The two morphologies i.e., Graphene and CNTs are focused here as represented in Figure 5.

Researchers across the globe have explored the different methods for the synthesis of carbon nanomaterials, which are illustrated in Table 3. There are new synthesis methods that includes surfactant-aided sonication, purification from the amorphous base, acid refluxing, air oxidation and vacuum-based pulverization.^46^ The following section is detailed about the CNT and Graphene-based nanomaterials.

## Carbon nanotubes (CNTs)

CNT is a rolled cylindrical tube form of graphene. It is available in the form of a single-layered rolled tube is a single-wall carbon nanotube (SWCNT), and multi-wall carbon nanotubes (MWCNTs). The presence of π-π interaction on the outer surface of CNT is responsible for its penetration through the biological barriers.^47^ They are surface modified or functionalized (covalent and noncovalent) for increasing biocompatibility.^48^

###  Case studies

Cancer ablation: Moon and colleagues investigated SWCNTs for PTT. *Ex vitro* NIR irradiation of formulated CNT-PEG conjugates showed a quite rapid temperature increase with high thermal energy dissipation. *In vivo* evaluation of conjugates on mice induced with HOK B cells obliterated the induced tumour. Additionally, it showed complete eradication of solid tumours without side effects, abnormality without recurrence of a tumour for a long time period compared to single CNT treatment and blank treatment. The 60 days *in vivo* treatment with CNTs on mice shown the absence of lesion and negligible tumour volume (comparison: treatment with plain CNTs). The biodistribution studies of the conjugates showed the excretion of SWCNT through the biliary pathway and resolved the issue of residual CNT toxicity.^49^

Glioblastoma: Eldridge and colleagues evaluated MWCNT conjugates for glioblastoma multiforme (on GBM/U87cells).^50^ They considered issues such as inaccessibility and risk to brain damage, penetration into blood-brain barrier, and resistance to conventional therapy while designing the CNTs. To overcome such issues, the authors modified CNTs with PEG and aqueous surfactant distearoyl-sn-glycero-3-phosphoethanolamine (DSPE) by conjugation. The surfactant conjugation enhanced the diffusion of CNTs through brain phantoms (simulated brain model). The irradiation of conjugates acquired ablation temperature, without heat shock response. In the treatment, irradiation of CNTs maximized intra-tumoral dissemination with heat generation and minimum cytotoxicity. The CNT treatment with laser showed highest ablation at 90 seconds compared to 60 seconds. Overall, the study was conducted as a pre-clinical insight for translation, with respect to delivery and investigation of cytotoxicity of CNTs and its efficacy for GBM. This product improved the diffusion of CNT in the brain-ECM-mimic model (Easy for understanding the response of heat resistant cells). It also revealed that a rapid and effective distribution of heat ablates the cells, reduced thermal diffusion, and heat conduction.^50^

## Photodynamic therapy using CNTs

###  Case studies

CNTs have also been explored for their use in photodynamic cancer therapy. Huang et al developed CNTs conjugated poly-amidoamine dendrimer for delivery of 5 amino-fulvic acid. The photoablation ability of conjugates was evaluated on MGC-803 cells (*in vitro*). Irradiation of CNTs for 10 minutes showed 90% cell viability. As the concentration of CNTs raised to 30 μM, almost all tumour cells were found dead upon comparison to the standalone group showing no ablation.^51^

A similar case study was reported by Wang et al. They comparatively evaluated conjugates of SWCNT-polyethyleneimine (PEI) and SWCNT-Polyvinylpyrolidone-K30 (PVPK30). Authors characterized CNTs in mice melanoma B16-F10 cells (PEI probes v/s PVP Probes). 10 days of the treatment with CNTs, showed a significant reduction in the tumour, confirmed with comet assay (DNA damage). The PEI containing CNTs damaged the tumour with the least volume (Relative volume 6.6compared to K30 7.7) compared to PVP-K30+ CNT.^52^

## Combined therapies with CNT

 Due to the versatility and loading capacity of CNTs, many anticancer agents have been evaluated for the combined CNT therapies. Few of the representative studies are put below where researchers have explored the efficiency of CNTs.

###  Case studies 

Breast cancer: A hybrid platinum-acridine anticancer agent (P3A1) containing MWCNT was developed by Fahrenholtz et al for breast cancer. The conjugates of the nanotube with P3A1 and polymer DSPE-mPEG were synthesized by one-step reduction and purified. The *in vitro* PTT evaluation of CNTs in MDA-MB-231 cells, showed an increase in temperature to 44ºC, after 60 seconds of exposure. CNTs killed breast cancer cells. Add on to this effect, the generated heat altered release of photosensitizer from CNT.^53^ The *in vivo* studies of CNT on mice cancer models (induced with MDA-MB-231 cells) showed that plain P3A1 ablated cancer cells, but could not stop the cancer progression post-treatment. The PTT activated P3A1+CNT, generated mild hyperthermia with cytotoxicity of CNTs. However, the combined therapy did not show cancer progression post-treatment. Overall, the investigation suggested that P3A1 + CNT conjugate is beneficial with combining PTT for intractable cancer.^53^

Development of Redox system for Cancer: Hou and colleagues developed a redox-sensitive system of SWCNT for chemo- PTT. The probe had hyaluronic acid (HA), DOX and gadolinium (Gd) within it. One set of probes was formulated by disulphide (ss) bonding. The *in vivo* evaluation of these conjugates on mice model (with MCF7 cells) showed an effective internalization into the MCF 7 (Michigan cell foundation/Breast cancer) cells, through HA receptor-mediated endocytosis. Endocytosis rapidly transported CNT conjugates to the cytosol. It improved the ablation capacity of the material, with the aid of IR irradiation showing a synergistic effect.^54^ The *in vivo* evaluation of the conjugate Gd/SWCNTs-HA-ss-DOX with laser (tumour volume ratio <1) on mice showed low relative inhibition rate (tumour volume ratio) compared to the other formulations (SWCNTs-HA-ss-DOX, SWCNTs-HA-DOX, Gd/SWCNTs-HA-DOX laser, Gd/SWCNTs-HA-ss-DOX showed tumour volume ratio more than 1.5). The therapeutic outcomes by these probes showed promising results for cancer therapy and diagnostics.^54^

## Graphene based nano-theranostics

Graphene is a graphite derivative made up of a one-atom-thick planar sheet of sp2-bound carbon. It forms a dense honeycomb lattice with a plane surface and high thermal conductivity. Graphene derivatives like graphene oxide (GO) and reduced graphene oxide (rGO) are explored for phototherapies. rGO has shown an enhanced absorbance in the NIR region, making it a better candidate for PTT application. Graphene can be synthesized by top-down and bottom-up methods. Top-down methods include micromechanical cleavage, electrochemical exfoliation, thermal or chemical reduction of GO and unzipping of CNTs (mentioned in Table 3).^21^ The bottom-up approaches are chemical vapor deposition and epitaxial growth on silicon carbide. Graphene formation depends on the growth of carbon molecules on certain substrates. Functionalization of graphene with different functional groups (epoxide, carbonyl, carboxyl, and hydroxyl) makes it water dispersible.

## Photothermal therapies with graphene

###  Case studies

Triple effect with graphene: Graphene is well explored for the treatment of multidrug-resistant cancer. Yang et al developed combined chemo + PTT to overcome the problem of low drug accumulation responsible for MDR. For overcoming low drug accumulation, they conjugated graphene containing C225 with cetuximab and epirubicin (EPI). These conjugates showed a targeted action with a triple effect in the cancer treatment. Triple effect prolonged survival of the subject (50 days). The nanoconjugates were investigated *in vitro* on glioma U87 cells. The in-vivo evaluation of conjugates suggested that they were nontoxic and specific for malignant cancers with effective heat absorption. The hybrid NT of anti-EGFR (C225) with EPI, targeted malignant glioma cells and inhibited cancer propagation. The irradiation of conjugate damaged DNA structure and burnt residual tumour cells.^55^ The *in vivo* treatment of Graphene+EPI+C225 conjugate with laser showed 100% survival compared to the groups administered with free EPI, treatment without laser and individual graphene conjugate of EPI. The 21days treatment also showed reduced tumour volume and weight (125 times less than hybrid without laser). In total, the conjugates synergistically targeted with a triple-treatment which completely cured the tumour. It also enhanced local drug concentration at cite (6.3-fold) and aided in overcoming the multi-drug resistance.^55^ This triple targeting ultra-effectively suppressed the tumour and prolonged mice survival.^55^

Li and colleagues successfully formulated multifunctional cisplatin conjugate [Pt (IV) complex- PEGylated GO] as a single platform containing a sensor for cancer cell apoptosis. The conjugate was investigated for combined thermal chemotherapy with real-time monitoring. An *in vivo* evaluation of conjugates (female mice/ 4T1 cells) showed an enhanced anticancer effect evaluated with the tumour volume [<1(Laser+ conjugate) v/s 1 (compared to plain conjugate)], high tumour targeting at elevated temperature, displayed a synergistic effect. Along with it, the therapy indicated complete tumour destruction without re-occurrence. In summary, the combined chemotherapy of platinum with GO as a single platform induced with hyperthermia showed numerous advantages namely precluding premature release, tumour-targeting, minimized systemic toxicity, efficient remote-control drug release. The platform showed great potential for clinical applications.^56^

## PDT using graphene

###  Case studies

Dong et al firstly reported the PDT with graphene. They double conjugated methoxy-PEG with Nano Graphene oxide. The photosensitizer zinc phthalocyanine was loaded on the NGO by hydrophobic interactions with p-p stacking with 14% drug loading (by weight). The graphene demonstrated significant cytotoxicity in MCF-7 cell lines in response to Xenon light radiation.^57^

The hypocrellin photosensitizers received limited attention, due to their low aqueous stability and poor solubility issues. Since hypocrellins are known for a high quantum yield of ROS, Zhou et al developed stable Nano-GOs of hypocrellin. Authors loaded hypocrellin B and hypocrellin A on GO nanosheet, by hydrogen bonding interaction. The graphene-conjugates effectively delivered them at the target. An *in vitro* PDT application of nanocomposites on HeLa, SMMC-7721, SGC-7901 and A549 cells marvellously damaged cancer cells compared to plain material.^58,59^

## Hybrid nano-theranostics

 Recently, in addition to the mono NTs, scientists have also explored the effect of hybrid NTs. Hybrid combinations are recent among the smart theranostics, composed of combined metal and carbon-based material for the purpose of theranostic application. They showed a super-effective cancer treatment.

###  Case studies

Combined nanostars with CNT: Zhu et al developed, a hybrid dispersion of conjugated GNS with MWCNTs. The individual and hybrid formulation were evaluated *in vitro* and *in vivo* on B16F10 cells. Hybrids showed improved PTT along with cell ablation compared to plain and standalone materials. Though the cell death was observed 80% with aid of laser but the viability was seen comparatively less with hybrids. The laser irradiation also showed the highest temperature gain in hybrid. The investigation proved the great potential of hybrid as a promising nano-platform for cancer therapy.^60^

Hybrid metal and carbon dot conjugated CNTs: Zhang et al developed probes of Fe_3_O_4_/carbon quantum dots SWCNTs for dual-mode target imaging in a triple mode therapy (Chemotherapy/PTT/PDT).^61^ CNT probes served as a carrier, a photothermal heater, ROS generator and a multimodal imaging probe.^61^ *In vivo* evaluation of hybrid probes (induced with the HeLa cell mice model) showed complete inhibition of cancer.^61^ The hybrid probe showed the highest temperature elevation compared to probes with an individual agent. Hybrid probe with high power laser elevated temperature up to 50ºC compared to low power. The volume of the tumour was observed lowest in triple combination compared to standalone therapy individually. The investigation concluded that a hybrid increased accumulation of conjugates in the malignant tumour tissues compared to the control group. It proved that probes are promising drug delivery for cancer required a drug targeting.^61^

## Challenges and limitations

 Although, NTs have showed promising outcomes, it is also associated with certain limitations and challenges.

In the case of GNPs, it easily forms an aggregate. They slowly degrade in the body. This phenomenon may cause accumulation in the liver and spleen. It might result in an inflammatory response.^62^ A major limitation for the GNP includes a low tumour binding capacity. In the therapy, GNPs requires high energy and a long time for activation.^62^ For the PTT with GNPs, it requires the operation with strict control on the laser irradiation. Otherwise, it may result in damage to the healthy tissue near tumour surroundings.^62^ The GNPs are expensive to manufacture.^62^ However, a high production cost for GNP is benefiting on another side by showing high effectivity and fewer side effects with reduced mortality. Moreover, treatment with GNPs also reduces the chance of hospitalization and decreases the personnel cost.^63,64^ The challenge with carbon-based materials includes a poor capacity to convert light to heat, slow degradation, low sensitivity, low accumulation at the site, non-specific biodistribution, poor circulation in blood and uncontrolled side effect of the released chemotherapeutic drug and high affinity towards the liver etc.^42-65^

## Toxicity parameters

GNPs are vastly explored in the NTs and regarded as safe.^65^ For GNPs, their toxicity depends on size, modification on the surface, circulation time in body and concentration etc. The surface charge also makes an impact on the product with its toxicity parameters.^66^ The GNPs more than 1.4nm in diameter are toxic due to the irreversible binding.^67^ The modification of GNP with the PEG molecule increases the hydrodynamic diameter of the probe which alters the pharmacokinetic and biopharmaceutical parameters. The cationic particles show higher toxicity, compared to hydrophobic and poorly dispersed particles (cleared easily).^67^ In case of size particles below 8nm are cleared off by the kidneys. The parameters like size, shape, functionalization and method for in-vivo administration, play an important role in the toxicity.^67^

Despite of these issues, product such as Aura-immune is already found success in the 1st phase of a clinical trial.^66^ The therapy employed using gold silica nano-shell is under investigation for the human trials (ClinicalTrials.gov, Identifiers: NCT02680535).^66-68^ GNPs have exhibited low toxicity and high chemical stability.^66^

In the case of carbon NTs, cellular toxicity to a human cell depends on the purity and synthesis method.^69^ The exposure time of the carbon material also plays an important role. A case study has showed that the concentration of CNT in the range of 0-0.1 µg/mL has no proven toxic effect on the human lymphocytes.^70^ In the case of CNTs, the structure MWCNTs may show less cellular toxicity than the SWCNT.^71^ Toxicity also depends on surface charge, shape, length, diameter, agglomeration, and purity. These are hard to keep consistent due to different preparation and purification procedures. They mainly influence the toxicity of CNTs.^72^

Most of the carbon-based nanomaterials are surface modified with hydrophilic material.^73^ It reduces the issue of low solubility which possibly cause the deposition onto human cell resulting due to improper dispersion.^74^ Such a process may reduce solubility which possibly causes the deposition onto human cells resulting due to improper dispersion.^74^ Overall for the carbon theranostics, toxicity depends on the purity, production parameters and the capacity to penetrate in the nucleus and cytoplasm.^73,74^

Moreover, the functionalization density of CNT is the main factor among toxicity considerations. Functionalization of CNT with oxygen depends on the refluxing time. The CNT of more than 20 µm (length) is responsible for granuloma, resulting in an inflammatory response. The blood circulation time is an important factor for CNTs. The biodegradable CNTs are less toxic than non-biodegradable ones.^75,76^

For the graphene-based material, pristine graphene shows dose-dependent toxicity to mice.^77^ The functionalization of graphene oxide with biocompatible coating by chemical reaction has reduced the harmful effects and *in vivo* toxicity.^78^ The toxicity of graphene quantitatively depends on chemical structure, defects, layering, size, and charge.^77,78^ Other factors influencing toxicity include dosing route, drug type, cell type and exposure time.^79^

To address the toxicity issues focus has to be kept on the evaluating effects of surface chemistry, shape and size of GNPs on their *in vivo* toxicity and biodistribution.^80^ For carbon-based materials, parameters which are required to monitor are physiochemical and structural characteristics, the dose and time of exposure, cell type, mechanism, residual catalyst and synthesis method.^80^ Additionally, there is a requirement to establish the long-term study of NTs related to biodistribution, circulation, translocation, metabolism, degradation, secretion and effects on the nervous system.^80,81^

## Conclusion

NTs have significantly improved the quality of clinical care and cancer treatment. Their features like predictive, preventive and personal medicine render an appropriate drug and dose to the patient at right time. The gold, carbon NTs, hybrids and combined therapies have addressed the precocious treatment. These are stable, biocompatible and specific for the treatment. The excellent key features of NTs are a reversal of resistance, stop on the cancer re-growth and immunity implementation etc. To improve the regulatory status and clinical translation, NTs need to be evaluated for long-term and short-term studies related to their *in vivo* effects. Simplification of synthesis by using a nano-pulverization approach, evaluation of stability and safety will aid the upscale of NTs from lab to manufacturing. Overcoming the challenges and limits will aid and open new opportunities for the development of novel and unique techniques for cancer theranostics.

## Acknowledgments

 The authors duly acknowledge Indian Council of Medical Research (ICMR), New Delhi and Manipal College of Pharmaceutical Sciences, MAHE for their support.

## Ethical Issues

 Not applicable.
